# Tracking Initial Fe(II)-Driven Ferrihydrite Transformations: A Mössbauer Spectroscopy and Isotope Investigation

**DOI:** 10.1021/acsearthspacechem.2c00291

**Published:** 2023-09-28

**Authors:** Drew Latta, Kevin M. Rosso, Michelle M. Scherer

**Affiliations:** † Department of Civil and Environmental Engineering/IIHR, The University of Iowa, Iowa City, Iowa 52242, United States; ‡ Physical Sciences Division, Pacific Northwest National Laboratory, Richland, Washington 99345, United States

**Keywords:** Fe(II)−Fe(III) interfacial electron transfer, ferrihydrite transformation, recrystallization, dissolution−reprecipitation, induction period, lag phase, labile Fe(III)

## Abstract

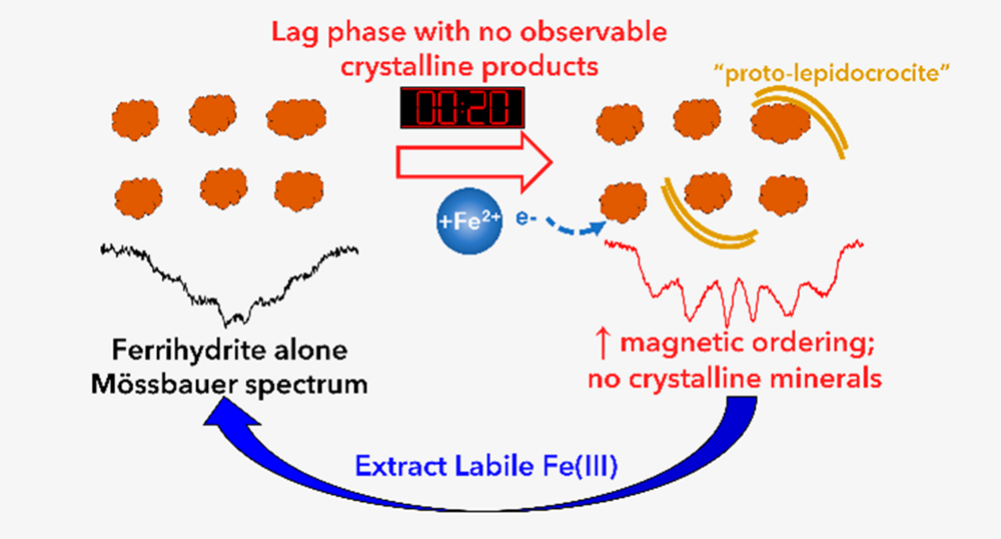

Transformation of nanocrystalline ferrihydrite to more stable microcrystalline Fe(III) oxides is rapidly accelerated under reducing conditions with aqueous Fe(II) present. While the major steps of Fe(II)-catalyzed ferrihydrite transformation are known, processes in the initial phase that lead to nucleation and the growth of product minerals remain unclear. To track ferrihydrite–Fe(II) interactions during this initial phase, we used Fe isotopes, Mössbauer spectroscopy, and extractions to monitor the structural, magnetic, and isotope composition changes of ferrihydrite within ∼30 min of Fe(II) exposure. We observed rapid isotope mixing between aqueous Fe(II) and ferrihydrite during this initial lag phase. Our findings from Mössbauer spectroscopy indicate that a more magnetically ordered Fe(III) phase initially forms that is distinct from ferrihydrite and bulk crystalline transformation products. The signature of this phase is consistent with the early stage emergence of lepidocrocite-like lamellae observed in previous transmission electron microscopy studies. Its signature is furthermore removed by xylenol extraction of Fe(III), the same approach used to identify a chemically labile form of Fe(III) resulting from Fe(II) contact that is correlated to the ultimate emergence of crystalline product phases detectable by X-ray diffraction. Our work indicates that the mineralogical changes in the initial lag phase of Fh transformation initiated by Fe(II)–Fh electron transfer are critical to understanding ferrihydrite behavior in soils and sediments, particularly with regard to metal uptake and release.

## Introduction

The nanocrystalline ferric iron hydroxide, ferrihydrite (Fh), is widely distributed in soils and sediments^1,2^ and plays an important role in controlling biogeochemical redox processes^3^ from carbon transformations^4,5^ to the fate and transport of a wide variety of contaminants, nutrients, and metals.^6−10^ Because of its nanocrystalline nature (i.e., 2–10 nm particles) and metastable properties, Fh slowly transforms (time scale of years) to more crystalline Fe(III) oxides, such as goethite (Gt) and hematite (Hm), under oxic conditions.^11,12^ However, under reducing conditions, the presence of dissimilatory iron reducing bacteria,^5,13^ reduced organic matter,^14−16^ and Fe(II)^17,18^ rapidly accelerate the rate of Fh transformation (time scale of minutes) to more crystalline product minerals.^16^ This rate acceleration has been termed “Fe(II)-catalyzed transformation”^19^ and has been observed for Fh as well as other metastable Fe(III) minerals such as schwertmannite.^20^ The Fe(II)-catalyzed transformation of Fh can yield a wider range of crystalline products beyond just Gt and Hm, including metastable green rusts and lepidocrocite (Lp) to magnetite (Mt).^17,18,21,22^

Several steps have been identified in Fe(II)-catalyzed Fh transformation, starting with the relatively rapid uptake of Fe(II) by the Fh surface followed by interfacial electron transfer (IET) from adsorbed Fe(II) to structural Fe(III).^23−25^ Injection of electrons from the surface results in mobile electrons within the Fh.^23−27^ These delocalized electrons can go on to reduce Fe(III) atoms at spatially separated surface sites, resulting in the re-release of Fe(II) into solution.^28^ It is this cycle of adsorption, electron transfer, and reductive dissolution that results in rapid mixing between aqueous Fe(II) and bulk Fh, leading to erasure of isotope contrast observed with Fe isotope tracers.^9,18,29−31^

While the major steps of Fe(II)-catalyzed Fh transformation are reasonably clear, the details of the initial steps that ultimately lead to the emergence of microcrystalline product minerals are not, particularly during the initial contact time with aqueous Fe(II) (<30 min).^30,32−34^ In most batch experiments, this “lag phase” or “induction period” can be defined as the time after the net Fe(II) uptake by Fh is effectively complete (typically <10 min) but before the appearance of XRD-detectable stable product minerals. One conceptual framework proposed for this initial stage is that the Fh transformation proceeds through a reactive intermediate phase (originally termed “reactive” Fh), presumably formed at the Fh surface during IET.^32^ Although this concept was invoked to principally accommodate this lag phase in kinetic modeling,^19,32^ recent work supports this concept by showing the accumulation of Fe(III) more labile than Fe(III) in Fh during this lag phase, which was identified using the Fe(III)-selective ligand xylenol orange (XO).^30^ The accumulation of this labile Fe(III) pool on the Fh surface to a critical concentration was shown to be correlated to the nucleation and growth of product phases and Fh dissolution.^33,35,34^ A recent study hypothesized that the observed labile Fe(III) intermediate produced from aqueous Fe(II) may be linked to nanoscale transformation products akin to the proto-Lp sheets observed by TEM that occur early in the lag phase.^36^ However, despite this hypothesis, little spectroscopic evidence exists to describe the nature of the labile Fe(III) pool formed at the Fh surface during the lag phase. Mössbauer spectroscopy is particularly useful here, as isotopes can be used to selectively probe different pools of Fe in Fh.

To gain further insight into the mechanistic processes occurring within the initial lag phase, paying particular attention to the role of the labile Fe(III) intermediate, we used Fe isotopes, temperature-dependent Mössbauer spectroscopy, and extractions to monitor the structural, magnetic, and isotope composition changes within ∼30 min of Fe(II) exposure. We focus our work on the spectroscopic nature and isotope composition of the Fe(III) phases forming from Fh during the initial lag phase of the transformation to more crystalline products. Our findings from Mössbauer spectroscopy indicate that a more magnetically ordered Fe(III) phase initially forms that is distinct from ferrihydrite and bulk crystalline transformation products. Using xylenol orange extractions, we further show that this magnetically ordered Fe(III) is removed with the labile Fe(III) and may be due to the extraction of nanoscale transformation products such as proto-Lp sheets. Our observations show changes in the physical characteristics of Fh solids during Fe(II) contact over very short time scales that support the hypothesis that at near-neutral pHs, redox-driven Fh transformation occurs through a dissolution–reprecipitation mechanism mediated by an Fe(III) pool that results from IET and may be linked to incipient product mineral phases.

## Materials and Methods

### Fe Stock Solutions

Naturally abundant Fe(II) (^NA^Fe), ^56^Fe(II) (99.92% ^56^Fe, Isoflex), and ^57^Fe(II) (97%, Cambridge Isotope Laboratories, Inc.) stock solutions were prepared by dissolving Fe(0) in 2.25 M HCl (trace-metal-grade) and diluting to a 100 mM total Fe concentration with deionized water. After dilution, the stocks were filtered with a 0.22 μm nylon filter to remove any remaining Fe(0).

To convert the ^56^Fe(II) in the stock solution to Fe(III), we used bulk electrolysis. Here, bulk electrolysis with a potentiostat (Pine Instruments AFCBP1 bipotentiostat) was employed to avoid problems with the Fenton-type production of Fe(II) that we have previously observed when storing Fe(III) solutions oxidized with hydrogen peroxide. Here, 90 mL of the 100 mM ^NA^Fe stock was placed into a bulk electrolysis cell (BAS, Inc., MF-1056) with a reticulated vitreous carbon working electrode and a platinum counter electrode contained in a fritted glass chamber containing 0.2 M HCl. Electrolysis was done under a constant potential of +0.81 V applied relative to a Ag|AgCl (saturated KCl) reference electrode. Electrolysis was periodically stopped to replace the 0.2 M HCl counter electrode chamber electrolyte, as indicated by compliance overload on the potentiostat resulting from the evolution of H^+^ to H_2_ (g). Electrolysis was considered complete when the current was stable and less than 1 μA and no Fe(II) (<0.5 μmol/L) was detected with 1,10-phenanthroline.

### Two-Line Ferrihydrite Synthesis

Following the procedures established in previous and related studies,^24,30,34−37^ two-line ^56^Fe-enriched ferrihydrite (^56^Fh) was precipitated by titrating 50 mL of the Fe(III) stock with 2 M NaOH to pH 5.0 followed by continued addition of 0.2 M NaOH to a pH of 7.2, taking care not to exceed the pH of 7.4 at any point during addition of NaOH. The Fh suspension was allowed to age overnight (∼20 h), and the pH was readjusted to 7.2 with 0.2 M NaOH. The naturally abundant Fe isotope ferrihydrite (^NA^Fh) was synthesized as above, using 100 mL of a 100 mM FeCl_3_·6H_2_O stock as the starting solution. The solids were centrifuged at 5000 × *g* and washed with deionized water three times. The solids were dispersed by sonication into 50 mL of deionized water. Finally, oxygen was removed by bubbling with N_2_ for 1.5 h, and the solids were placed in an anoxic glovebox (93% N_2_/7% H_2_ gas) and allowed to further degas for 48 h. The final Fe concentration of the Fh stock suspensions was ∼200 mM.

### Ferrihydrite Transformation Experiments for Mössbauer Spectroscopy

Ferrihydrite transformation experiments for Mössbauer analysis were conducted in 18 mL of a 10 mM MOPS buffer containing a 10 mM NaCl electrolyte set to a pH value of 7.0 and varying concentrations of ^56^Fe(II). Experiments were conducted by first adding the required aliquot of ^56^Fe(II) to 17 mL of the buffer and taking a subsample to measure the initial Fe(II) concentration. Then, 2.9 mL of the ^NA^Fh suspension was centrifuged and resuspended in 1 mL of buffer and spiked into the ^56^Fe(II)-containing buffer. As needed, the pH was adjusted back to 7.0 using 0.2 M NaOH. The ^NA^Fh suspension was allowed to react for 5 min and then was filtered to recover the solids using a 0.22 μm nylon filter. The total elapsed time, including the time the solids were exposed to Fe(II) during filtration, was 20 min. Transformation experiments where ^57^Fe(II) was the source of the Mössbauer-active isotope were also conducted with ^56^Fe-enriched-Fh (^56^Fh), where a ^57^Fe(II) stock was added to 13.8 mL of a buffer. Subsequently 1.25 mL of the ^56^Fh stock was centrifuged and resuspended in 1 mL of buffer to achieve ∼10 mM Fe(III) in the final reactor. As described above, aliquots were taken for the initial and final Fe(II) concentration, and the pH was adjusted, as needed, to achieve pH 7.0.

We note that most studies investigating Fh transformation have employed organic “Good’s”-type pH buffers, including MOPS,^31,36,38^ PIPES,^17,30,34,35,37^ and HEPES.^24^ Importantly, as is further discussed in this study, similar trends in chemical processes such as electron transfer, atom exchange, and the production of label Fe(III) are observed in these studies. However, some studies have observed an influence of MOPS and other organic buffers on redox reactions and Fe(II) uptake.^39^ On the other hand, we note the significant decrease of the pH in unbuffered systems^30^ and the well-known effect of carbonate, as an alternative buffer, in mediating transformation products and pathways.^17,34^

### Xylenol Orange Extractions

Xylenol orange (XO) extractions of Fe(II)-reacted were conducted as previously described in Sheng et al.^30^ Reactors containing 15 mL of Fh and the ^56^Fe(II) or ^57^Fe(II) suspension were reacted for 20 min, 1.5 mL of a 5 mM XO solution was added, and the pH was adjusted to 5.6. Solids were extracted for ∼2 min and captured on a filter, as described above. The XO–Fe(III) complex was quantified by diluting ten times into 40 mM HCl and measured at 560 nm in a spectrophotometer.

### Ferrihydrite Isotope Exchange Experiments

Isotope exchange experiments between ^NA^Fe ferrihydrite (^NA^Fh) and ^57^Fe(II) were done in a similar way to that described above in 15 mL of a 10 mM MOPS and 10 mM NaCl buffer (pH 7.0). An aliquot of 150 μL of 100 mM ^57^Fe(II) was added to the buffer, and the initial Fe(II) concentration was measured. To initiate the reaction, an aliquot of 1 mL of a ∼200 mM Fh suspension was added to the Fe(II) and buffer solution. Each time point measured was a set of triplicate reactors. Reactors were placed on an end-over-end rotator for 20 min, 2 h, and 21 h.

Samples for aqueous Fe(II) and the aqueous isotope composition at each time point for the three vials were collected by filtering 2 mL of the suspension into a vial with 50 μL of trace-metal-grade 6 M HCl to preserve the Fe(II) from inadvertent oxidation. A separate 2 mL aliquot was centrifuged at 9000 × *g*, and the supernatant was carefully removed. Sorbed Fe(II) was extracted from the pellet of Fh by adding 2 mL of a 10 mM PIPPS (piperazine-N,N′-bis(3-propanesulfonic acid)) buffer at a pH of 3.5. Finally, the remaining solids were again separated by centrifugation at 9000 × *g*, the PIPPS extract was removed and filtered into a separate vial, and the residual solids were dissolved in 2 mL of 6 M HCl. Samples for analysis of the Fe isotopes of the aqueous phase and PIPPS extraction were diluted 100-fold into 0.1 M HCl (trace-metal-grade) for ICP-MS analysis. Samples of the residual solids were diluted 500-fold for ICP-MS analysis.

### Ferrihydrite Dissolution

Ferrihydrite dissolution experiments were conducted on triplicate reactors constructed as described above and based on previous work by Zhou et al.^25^ The reactors were 25 mL Oak Ridge centrifuge tubes with an O-ring seal. Fh was contacted with Fe(II) for 20 min and subjected to centrifugation at 7500 × *g* for 5 min outside the anoxic glovebox. The total time to return the solids to the glovebox and remove the supernatant was 42 min. After the supernatant was decanted, filtered (0.22 μm nylon), and retained for analysis, 15 mL of a 10 mM PIPPS buffer with a pH of 3.5 was added, as described above, to extract Fe(II). The PIPPS buffer extraction was done for 20 min, and again the solids were separated by centrifugation at 7500 × *g* for 15 min. The solids were separated, and the supernatant was filtered and retained for analysis. Finally, 15 mL of 0.1 M HCl (trace-metal-grade) was added to the Fh solids, and samples were taken at regular intervals for Fe and isotope analysis. To separate the nanoparticulate Fh solids from the acidic suspension, a 500 μL aliquot of the suspension was placed in a 10 000 Da centrifugal filter (VWR, Inc.) and centrifuged at 9000 × *g* for 5 min. The liquid was retained for isotope analysis and Fe content.

A second dissolution measurement was made on Fh exposed to Fe(II) for 2 h followed by the above steps of PIPPS extraction and 0.1 M HCl addition, except after 60 min of dissolution and little change in the amount of Fe dissolved between 30 and 60 min, the solids were separated for a sequential extraction by 1 M HCl (trace-metal-grade) by centrifugation at 10 000 × *g* for 10 min. After the 0.1 M HCl extraction, the supernatant was removed and 15 mL of 1 M HCl was added for 20 min, and the solids were again centrifuged at 10 000 × *g* for 10 min. The supernatant was recovered, filtered, and saved for later analysis. The remaining solids were digested by 1:1 HCl:deionized water overnight.

### Analytical Measurements

Concentrations of Fe(II) were measured using 1,10-phenanthroline, and total Fe was measured by the reduction of Fe(III) to Fe(II) with hydroxylamine hydrochloride.^40^

Mössbauer spectra were collected on filtered (0.22 μm pore size nylon membranes) samples that were sealed between two layers of polyimide (Kapton) tape. Spectra were recorded in a variable temperature cryostat (Janis Research) using a constant acceleration drive waveform Mössbauer spectrometer (SEECo, Inc.) and a 50 mCi ^57^Co(Rh) source. All data were calibrated relative to α-Fe at room temperature. Mössbauer spectral fitting was done with the program Recoil^41^ using extended Voigt-based lineshapes.^42^

Inductively coupled plasma mass spectrometry (ICP-MS) data for Fe isotopes were collected using an Agilent 7900 quadrupole ICP-MS instrument (Agilent Technologies, Inc.). Argide polyatomic interferences (e.g., ^56^[ArO]^+^ and ^54^[ArN]^+^) were removed with a collision cell containing 100% He at a flow rate of 5 mL/min. Samples were diluted to approximately 500 ppb of Fe in 0.1 M HCl for analysis. Internal standards of 10 ppb ^59^Co and ^89^Y were introduced into the instrument using a second channel on the peristaltic pump and mixed with the sample using a tee-piece. Iron isotope mole fractions (*f*) were calculated by dividing the counts per second (cps) of isotope *n* by the sum of the total iron isotopes’ cps values, as given by
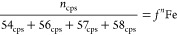
Because ^58^Fe is has an isobaric interference by ^58^Ni, ^60^Ni was monitored during analysis and mass 58 counts were corrected for the presence of Ni. Similarly, ^54^Fe was corrected for the presence of ^54^Cr by monitoring for ^52^Cr and correcting the mass 54 counts.

### Calculation of Incremental ^57^Fe Fraction in Each Extraction

To understand the iron isotope content of the Fh dissolved over the course of the dissolution experiments, the incremental isotope composition of each newly dissolved portion of the Fh was calculated during the Fh dissolution. These data were used to simulate sequential extractions where the solids were removed from each extraction step from dissolution data. To do this, first, the mass of ^57^Fe (^57^Fe_*i*_) was calculated from the measured fraction of ^57^Fe in the *i*th extraction step (^*i*^*f*^57^Fe) and the mass of Fe in the extraction (*m*_Fe_^*i*^) by eq 1:
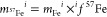
1For time steps of the 0.1 M HCl dissolution after the first time step (step 0), the incremental mass of ^57^Fe in that extraction step (^57^Fe_*i*_) was calculated as if a sequential extraction was done (eq 2). Using the incremental mass of ^57^Fe in each step, the fractional ^57^Fe content of the solution in each newly dissolved portion of Fh (Δ^*i*^*f*^57^Fe) was calculated from the following equation:
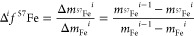
2where Δ*m*_^57^Fe_^*i*^ and Δ*m*_Fe_^*i*^ are the incremental masses of ^57^Fe and total Fe in the dissolution step, respectively, and *m*_^57^Fe_^*i*–1^ – *m*_^57^Fe_^*i*^ and *m*_Fe_^*i*–1^ – *m*_Fe_^*i*^ are the differences in mass of ^57^Fe and total Fe, respectively, from the current step and the previous step of dissolution.

### Safety Statement

No unexpected or unusually high safety hazards were encountered in this work.

## Results and Discussion

### Fe Mixing between Aqueous Fe(II) and Ferrihydrite

**Figure 1:**
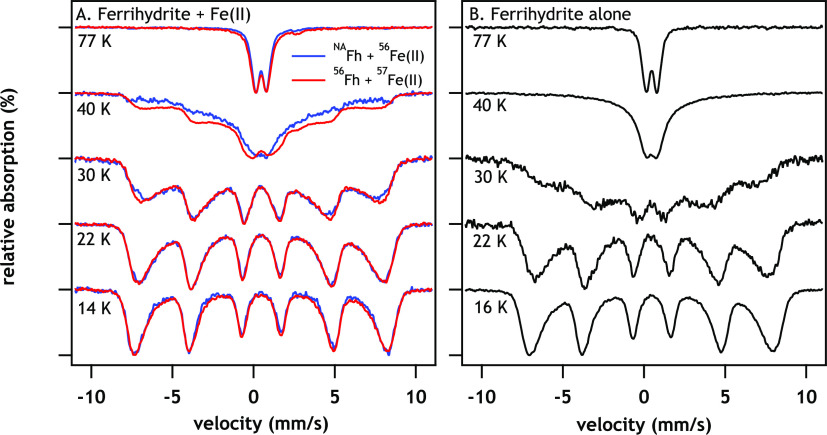
Mössbauer spectra of (A) an overlay of ^NA^Fh + ^56^Fe(II) (blue traces) with ^56^Fh + ^57^Fe(II) (red traces) after reaction with Fe(II) for approximately 20 min and (B) Fh in buffer. Data for each sample were collected from 14–16 to 77 K in the spectrometer. Conditions: ∼1 mM Fe(II), 6–8 mM Fh–Fe(III), 10 mM MOPS, and 10 mM NaCl at pH 7.0.

To evaluate the initial lag phase leading to rapid Fh transformation, we conducted experiments with isotopically labeled Fe(II) solutions and Fh using temperature-dependent Mössbauer spectroscopy within the first 20 min of contact (for solution data, see Table S1). We reacted Mössbauer-invisible ^56^Fe-ferrihydrite with ^57^Fe(II) for ∼20 min and collected the Mössbauer spectra as a function of temperature (Figure 1a, red spectra). Electron transfer between the ^57^Fe(II) and the underlying ^56^Fh results in the formation of a doublet having Mössbauer spectral parameters of Fe(III) at 77 K (center shift (CS) = 0.47 mm s^–1^, quadrupole splitting (QS) = 0.71 mm s^–1^, Table S2), consistent with previous experiments.^24^ The crystalline product minerals remained below detection by powder X-ray diffraction over this ∼20 min time frame (Figure S1). The Fh-like Fe(III) feature in the Mössbauer spectra changes between a doublet and a sextet at temperatures from ∼15–77 K. The change in the ^57^Fe(III) doublet to a sextet is a result of the magnetic ordering of ^57^Fe(III) at lower temperatures. Our observations with Mössbauer spectroscopy and XRD suggest that templated growth of a Fh-like ^57^Fe(III) mineral occurs when ^56^Fh is reacted with ^57^Fe(II), as has been previously observed.^23−25^

We then performed the same experiment except with the isotope labels switched, such that ^57^Fe was initially present in the bulk Fh (i.e., ^NA^Fh reacted with ^56^Fe(II)). We observed that temperature-dependent Mössbauer spectra were remarkably similar to the spectra observed with reversed isotopic labeling (Figure 1a, blue spectra). Hence, regardless of whether the ^57^Fe started in the solution or the bulk Fh, the spectra were nearly identical at 30, 22, and 14 K, with only slight differences observed at 77 and 40 K. The overlapping spectra provide compelling evidence that the Fe(III) product resulting from Fe(II)–Fh contact and IET is similar regardless of the starting location of the ^57^Fe. The similarity of the spectra indicates that rapid mixing of the Fe atoms is likely occurring, consistent with previous work.^29,31^

**Figure 2:**
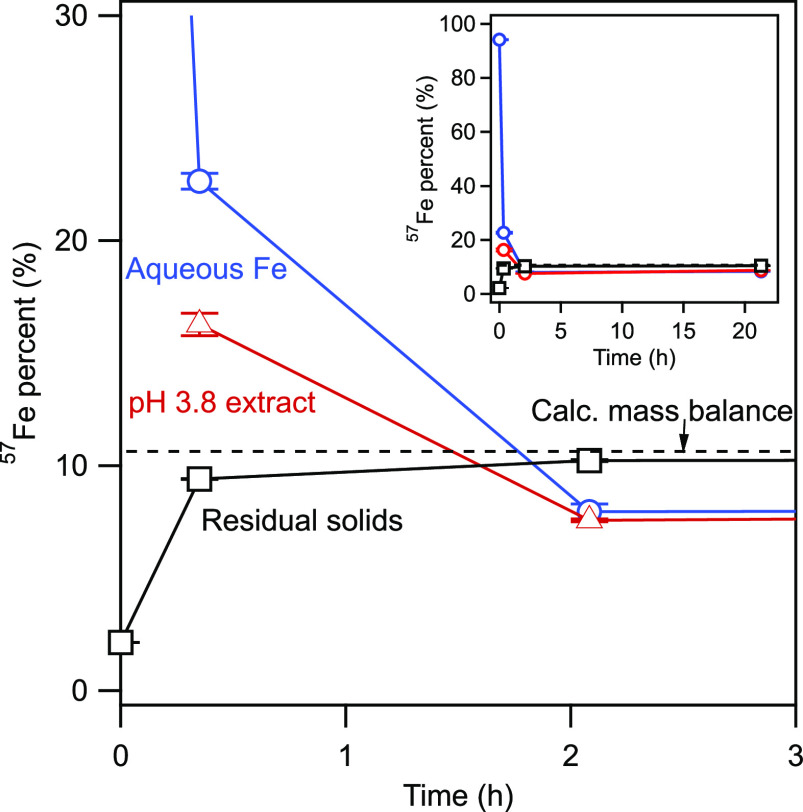
Percent ^57^Fe in aqueous Fe(II), extracted Fe, and residual Fh and product solids over time. The horizontal dashed line is the calculated, completely mixed ^57^Fe value of 10.6%. Inset: data plotted over the full range of 0–21 h. Conditions: 1 mM ^57^Fe(II), 10 mM Fh–Fe(III), 10 mM MOPS, and 10 mM NaCl at pH 7.0. Values for the data points represent the mean of triplicate reactors.

To confirm and quantify Fe atom mixing between the bulk Fh and the solution, we tracked Fe isotope movement between 1 mM ^57^Fe(II) in the fluid with ^NA^Fh over time by taking samples at 20 min, 2 h, and over ∼1 day (21 h) (Figure 2). (Fe(II) sorption equilibrium was attained within the first ∼20 min of contact.) We observed a rapid decrease in ^57^Fe(II) in solution over 20 min followed by a slower decrease toward and below the completely mixed line (10.6% ^57^Fe/Fe_total_) by 2 h of reaction, with little ensuing change by approximately 1 day. As expected, conversely, the isotope composition of the solid phase increased toward the completely mixed line. A pH 3.5 PIPPS buffer extraction^25^ targeting sorbed Fe(II) revealed that at 20 min, the predominant isotope composition of extracted Fe(II) (88.8% Fe(II)) was between that of the aqueous phase Fe(II) and the residual solids, consistent with the significant incorporation of ^57^Fe into the bulk of the Fh.

Both the isotope tracking experiments and Mössbauer spectra suggest rapid mixing of Fe atoms between the Fh bulk and the solution phase within the first 20 min of contact. The rapid mixing results in Mössbauer spectra that are nearly identical regardless of the original source of the ^57^Fe-labeled iron, and the isotopic compositions in the solid and solution rapidly approach one another during this lag phase of the transformation process. Rapid mixing of Fe isotopes from the solution to the bulk of the solids, and vice versa, suggests that significant dissolution of the Fh occurs, enabled by IET, consistent with the dissolution–reprecipitation mechanism which has been previously proposed for Fh transformation.^30,34,35^

### Magnetically Ordered Fh Phase

To gain further insight into changes to the Fh during the lag phase, we compared the isotope-labeled Mössbauer spectra of Fe(II)-reacted Fh with those of ^NA^Fh alone (with no added Fe(II)) (Figure 1). We observed Fe(II) in both labeled experiments, as expected, which arises from IET between Fe(II) and Fh.^24,25^ Importantly, Fe(II)-reacted Fh magnetically orders at a higher temperature than that of ^NA^Fh alone. The onset of magnetic ordering is most readily apparent at 40 and 30 K, but incipient magnetic ordering occurs in the ^57^Fe(III) near 77 K. The change in Fh spectra from 77 to 15 K is due to the change from superparamagnetic to antiferromagnetic magnetic behavior and is called the Mössbauer-derived superparamagnetic blocking temperature (*T*_B_^M^).^43^ Looking closely at the 30 K spectrum, the ^NA^Fh alone spectrum is a broad, collapsed sextet that shows the initial signs of magnetic ordering (Figure 1b) with an average hyperfine field of 28.6 T (Table S2). However, after only 20 min of reaction with Fe(II), the spectrum of the Fe(II)-reacted Fh is more ordered than the Fh alone. After the short exposure of Fh to Fe(II), the hyperfine fields of the Mössbauer spectra increased to 34.5 and 34.9 T for ^NA^Fh reacted with ^57^Fe(II) and ^56^Fh reacted with ^57^Fe(II), respectively (Table S2), and are consistent with the visual observation of an increased magnetic ordering of the Fh at 30 K. At lower temperatures, we also observed a larger hyperfine field splitting in the Fe(II)-reacted Fh spectra at 22 and 17 K than the ^NA^Fh spectra (Figure 1 and Table S2). The addition of Fe(II) also leads to an increase in the *T*_B_^M^ from approximately 50 K in ^NA^Fh alone to 60 and 70 K in ^NA^Fh reacted with ^56^Fe(II) and ^56^Fh reacted with ^57^Fe(II) (Figure S2), respectively, consistent with the increase in the hyperfine field after reaction with Fe(II).

Our finding of increased magnetic ordering of Fh after reaction with Fe(II) makes it clear that the physicochemical characteristics of the Fh assemblage change during this early stage of initial contact with Fe(II), prior to the emergence of crystalline products. This is particularly relevant to conclusions in recent prior studies that invoke a “reactive Fh” intermediate phase^32^ during this routinely observed lag phase of transformation. Our finding is furthermore reminiscent of our previous work on the Fe(II)-catalyzed transformation of natural organic matter–Fh coprecipitates, where IET and ^57^Fe atom exchange occurred with an increase in the magnetic ordering of NOM–Fh^25^ but without the formation of crystalline products over 28 days.

**Figure 3:**
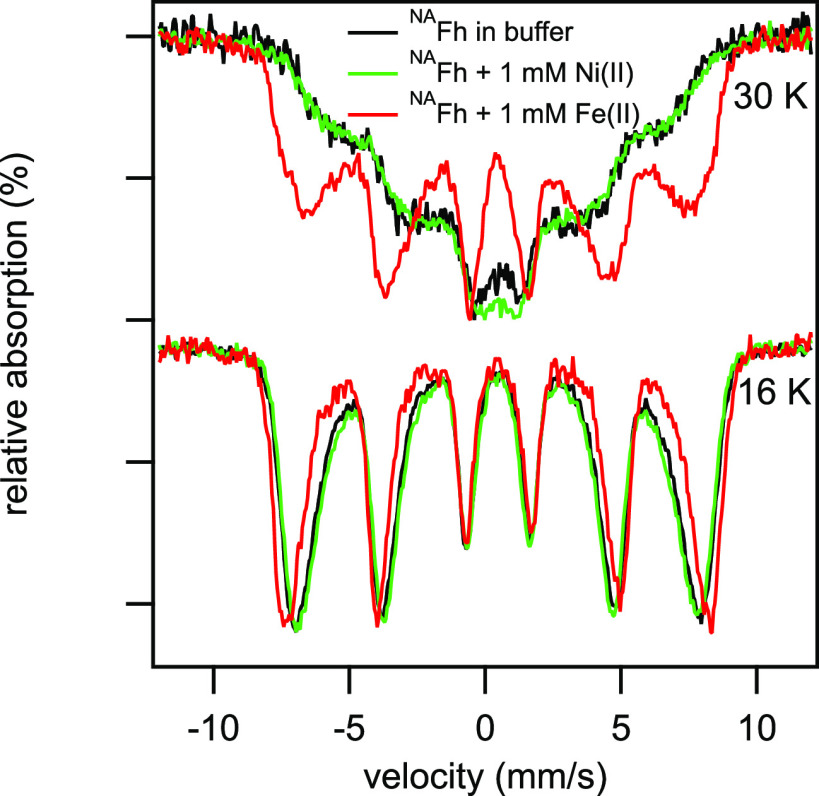
Comparison of ^NA^Fh alone in a MOPS buffer with ^NA^Fh reacted with ^56^Fe(II) and with ^NA^Fh reacted with 1.1 mM Ni(II). Conditions for ^NA^Fh + ^56^Fe(II): 10 mM MOPS, 10 mM NaCl, pH 7.0, Fe(II)_sorbed_ = 398 μmol/L, and [Fh] = 8.53 mM. Exposed to Fe(II) for 20 min. Conditions for ^NA^Fh + Ni(II): 10 mM MOPS, 10 mM NaCl, pH 7.0, [Ni(II)]_0_ = 1.1 mM, and [Ni(II)]_sorbed_ = 0.23 mM. Exposed to Ni(II) for 20 min.

Our results from both Mössbauer isotope experiments suggest that the transformation of Fh by Fe(II) to more crystalline Fe(III) oxides proceeds through a more magnetically ordered phase or mixture of phases. Given the sensitivity of Mössbauer measurements to a range of physical characteristics of the sample, a number of possible explanations for this observation exist. For example, the increase in the magnetic ordering of Fh and intermediates during exposure to Fe(II) could be explained by increases in aggregation,^44,45^ crystallinity, and/or particle size.^25,46^ To explore whether Fh aggregates when a divalent metal, such as Fe(II), sorbs, we reacted 1.1 mM Ni(II) with Fh and compared it to the Mössbauer spectrum of ^NA^Fh in buffer (Figure 3). We used redox-inactive Ni(II) to rule out a change in the surface charge or ionic strength-driven aggregation. Nickel uptake by Fh was similar to Fe(II) uptake (Table S1). The Mössbauer spectrum of Fh reacted with Ni(II) was indistinguishable from Fh suspended in buffer alone, suggesting that the observed change in the Mössbauer spectra during Fe(II) contact is not due to a change in the aggregation state of the Fh particles. While we cannot rule out a change in crystallinity or particle size as the cause of the magnetic ordering of the Fe(II)-reacted Fh, XRD patterns measured here and in our past work^25^ did not show any evidence for the formation of a more crystalline Fh, nor has any significant change in Fh surface area been observed under similar conditions over 4 h.^34^

Another potential explanation for the increase in Fh magnetism after exposure to Fe(II) is a decrease in uncompensated surface or bulk spins as a result of Fe(II)–Fh IET. The superparamagnetic to magnetic (antiferromagnetic) ordering behavior of two-line Fh has been related to changes in the number of disordered Fe atoms at the surface of the Fh particle.^43,47,48^ Computational modeling further suggests that tetrahedral Fe(III) in Fh may be present, in part, to relieve magnetic stress within the structure.^49^ Previously, we observed reduced Gt surface magnetism in synchrotron X-ray magnetic circular dichroism (XMCD) spectra after reacting Gt with Fe(II) and ascribed the change to the filling of vacancies during Fe(II)–Gt IET.^50,51^ It is therefore possible that the filling of vacancies at the surface, either by structural rearrangements that minimize defects or tetrahedral Fe(III) or by dissolution–reprecipitation, could explain our observations in the Mössbauer spectra. If the filling of vacancies is responsible for the increased magnetism, this would be consistent with the previous suggestion that near-isopotential structural states exist in the Fh system.^52^

A final explanation we can envision is that poorly crystallized but more periodically ordered phases are nucleating during the lag phase, such as the single-layer lamellae reminiscent of individual sheets of Lp recently observed by transmission electron microscopy (TEM) during this early stage at pH 7.0 (∼0.75 h)^34^ and over longer periods at pH 6.0.^33^ It has been shown that poor crystallinity in Lp can decrease the magnetic ordering temperature from the Neél temperature of 77 K to between 40 and 30 K, with hyperfine fields of ∼44 T.^53^ The Mössbauer parameters we observed are similar to those of Lp and suggest that poorly crystalline Lp could be present, as nanocrystalline Lp has Mössbauer spectral and temperature-dependent properties similar to those of two-line Fh.^53^ Even though we did not observe Lp in the XRD, small quantities and low crystallinity would likely preclude its detection by XRD, as has been observed for the discrepancy between μ-XRD results and TEM images at early times.^34^ We did, however, observe nanometer-sized thin and platy particles in SEM images (Figure S3) formed after 20 min of reaction with Fe(II) that are consistent with the formation of individual nanosheets of Lp and which were absent in the Fe(II)-free controls. These particles have considerable defects and porosity or are electron-translucent (Figure S3 and Discussion).

### Extraction of Surface Fe(III) Formed by Fe(II)–Fh IET by Xylenol Orange

Previous studies have shown that a more chemically labile form of Fe(III) than the Fe(III) in Fh emerges on the Fh surface upon initial Fe(II) contact, as assessed by XO extraction, and that this labile Fe(III) acts as an intermediate to crystalline product formation.^30,35,37^ Here we explored the extent to which these proposed changes to the Fh surface are consistent with the changes that we observe in the Mössbauer spectra. We conducted XO extraction of the Fh after 20 min of reaction and measured the Mössbauer spectra. We observed similar amounts of Fe(III) in the XO extracts (0.191–0.267 mmol L^–1^ Fe(III), Table S1) to those previously reported^35,37^ and estimate that this is about 3% of the total Fe.

**Figure 4:**
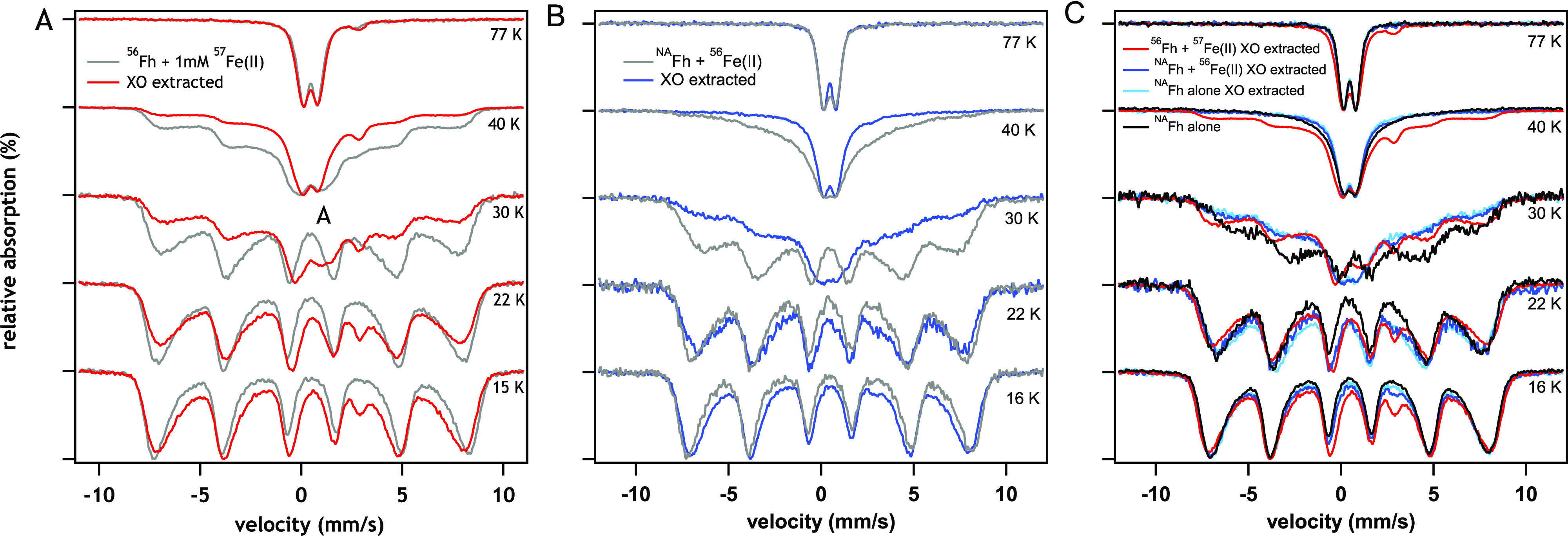
(A) Mössbauer spectra of ^56^Fh reacted with 1 mM ^57^Fe(II) before (gray spectra) and after extraction with XO (red spectra). (B) Mössbauer spectra of ^NA^Fh reacted with 1 mM ^56^Fe(II) before (gray spectra) and after extraction with XO (blue spectra). (C) XO-extracted Fe(II)-reacted spectra (as in A and B) compared with ^NA^Fh in buffer alone (black) and XO-extracted ^NA^Fh reacted in buffer alone. Reaction time was 20 min prior to a short XO extraction (∼2 min).

We measured Mössbauer spectra of Fh reacted with Fe(II) after XO extraction for both the ^56^Fh reacted with ^57^Fe(II) and ^NA^Fh reacted with ^56^Fe(II) (Figures 4a and 4b). The spectra in both cases show a decrease in the blocking temperature (*T*_B_^M^, Figure S2) and a decrease in the hyperfine field at ∼15 K of 41 T (Table S2) down from 44 T before extraction. Note that, for comparison, XO extraction of ^NA^Fh unreacted with Fe(II) resulted in little change in the Mössbauer spectra before and after XO extraction (Figure 4c) and a small (∼3 K) decrease in *T*_B_^M^, consistent with the relatively insignificant amount of Fe(III) removed by XO in the absence of IET between Fe(II) and Fh. For the Fe(II)-reacted Fh, both XO-extracted Mössbauer spectra are similar to those of ^NA^Fh alone, despite exposure to Fe(II). This strongly suggests that XO extraction removes the Fe(II)-catalyzed labile Fe(III) product that magnetically orders at higher temperatures, leaving behind Fh bearing its original characteristics. The result is somewhat surprising as only up to 3% of the total Fe is removed by XO extraction. However, a large change to the Mössbauer spectral parameters and blocking temperature due to a small chemical change is not entirely unexpected, and significant changes in Mössbauer or magnetic properties are observed in Fe oxide core–shell systems^54^ due to the presence of other magnetic solids^55^ and even due to adsorbed cations.^56^

Such a large change in the spectra with such a small amount of Fe(III) extracted is conceptually consistent with the selective extraction of the minor constituent Lp-like lamellae by XO. Exposure of just two octahedral Fe(III) layers of Lp to solution at the surface of the transforming Fh particles^34^ might provide easy access for XO complexation and extraction of Fe(III), potentially suggesting preferential dissolution of the high surface area, high surface exposure Lp-like lamellae observed in TEM studies and here in SEM images (Figure S3). Preferential removal of these more periodically ordered sheets and lamellae could readily explain the reversion of the Mössbauer spectra to that of unreacted Fh and would be consistent with the small amount of Fe(III) dissolving from Fh that has not been reacted with Fe(II). Therefore, we speculate that these Lp-like lamellae and the labile Fe(III) species are one and the same, representing the initial product of Fe(II)–Fh contact during the lag phase and leading ultimately to the emergence of XRD-detectable crystalline Fe(III) oxide product phases.

### Fe Isotope Gradients during Fe(II)-Catalyzed Transformation

Given the collective observations made during the lag phase, we designed experiments using ^NA^Fh/^57^Fe(II) focused on the subsequent period occurring between 0.5 and 2 h to examine the transformation products and isotope composition of the solids further in time. We observed no crystalline products after ∼30 min of reaction, but by 2 h, Lp (28% by Mössbauer spectral fitting) and a trace of Gt (8%) were present, with the balance as Fh (68%) (Figure S1). Given that by 21 h of reaction the relative amount of Gt increases (Figure S1), these results are consistent with previous works using an FeCl_2_ solution, in which growth of Lp is observed first followed by the replacement by Gt.^17,18,29,34^

As expected, the rapid isotope mixing between Fh and aqueous Fe(II) seen in the lag phase continues into this subsequent stage during the emergence of more stable Fe oxides (Figure 2).^18,29,31^ After 2 h, the PIPPS-extracted Fe(II) was indistinguishable from that of the aqueous phase (Figure 2). Although a rapid approach to isotopic equilibrium is consistent with previous reports on this system, complete erasure of the isotope contrast within 2 h is faster than previously indicated.^18,29,31^

We then sequentially dissolved and extracted the Fe(II)-reacted solids using acid extractions.^25^ During extraction and dissolution, we measured the amount of Fe dissolved and the isotope composition of the solution at the end of each time step and used those measurements to calculate the mass of ^57^Fe released to the solution during that time step (described in Materials and Methods).

**Figure 5:**
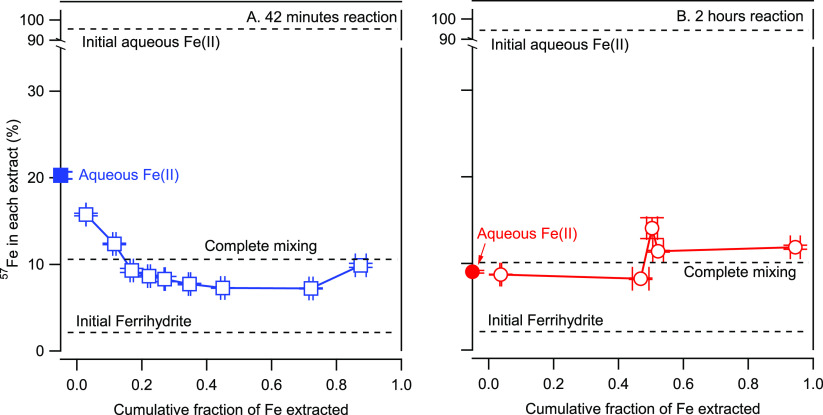
Percent of the ^57^Fe isotope in each fraction of Fe extracted from Fh after reaction with Fe(II) for (A) 42 min and (B) 2 h. Data are shown for Fh reacted with Fe(II) for 42 min and 2 h under similar conditions as those in Figure 1. The complete mixing lines are the calculated mass balance of percent ^57^Fe (10.5% and 10.1% for 30 min and 2 h, respectively) from the initial Fe(II) and Fh concentrations and their isotope compositions. Values for data points represent triplicate reactors. Conditions: 1 mM ^57^Fe(II), 10 mM Fh–Fe(III), 10 mM MOPS, and 10 mM NaCl at pH 7.0.

After 42 min of reaction with Fe(II), the isotope composition of the aqueous phase decreased from 94% ^57^Fe to 21% ^57^Fe (Figure 2). The initial extraction contained 15.7% ^57^Fe, indicating the near equilibrium of the sorbed Fe(II) and surface Fe(III) with aqueous Fe(II), similar to what has been observed with NOM–Fh coprecipitates and Gt.^25,57,58^ Further dissolution of the Fh revealed a decrease in the ^57^Fe content of the second and third aliquots (12.4% and 9.3%) followed by a gradual decrease to 7.2% ^57^Fe when 72% of the Fh was dissolved. In all cases, the later-dissolving aliquots were more enriched in ^57^Fe than the original solid (2.12% ^57^Fe) but had a lighter isotope composition than expected if complete mixing occurred (fully mixed, 9.56% ^57^Fe; dashed lines in Figure 5). The extractions clearly reveal a gradient of the ^57^Fe distribution through the reacted Fh particles. Furthermore, the residual solids in the final extract (9.89%) had a higher ^57^Fe content than the penultimate extract, suggesting that the solids dissolved at the end of the extraction were enriched in ^57^Fe.

In contrast to the isotope gradient developed after 30 min of reaction, after 2 h of reaction the aqueous phase and the first two extracts of the solids had similar ^57^Fe values (Figure 4, red trace), indicating that the gradient between the enriched ^57^Fe values in the first aliquots of extracted Fe was erased. While we are unable to localize where in the Fh particles and products the ^57^Fe is dissolved from, it is reasonable to assume that the initially dissolved Fe comes from the surface and further extractions come from deeper within the particles. We furthermore assume, though inconsequential to the interpretation, that the Fh surface is preferentially sampled more than the less soluble Lp and Gt products. The overall isotopic evolution is consistent with the finding established by Mössbauer measurements that a substantial but incomplete amount of mixing between Fe(II) and Fh occurs within 30 min and prior to the formation of XRD- and Mössbauer-detectable crystalline Fe(III) minerals.

At reaction durations of 2 h, where Lp and Gt products become XRD-detectable, the isotopic composition of the solution Fe “overshoots” the system’s isotopic mass balance (i.e., equilibrates to a somewhat ^57^Fe-deficient composition) (Figure 5), suggesting additional processes governing the distribution of this tracer. Specifically, we observed the aqueous Fe(II) (9.04% ^57^Fe) and initial PIPPS and HCl extractions of the 2 h solids having measured ^57^Fe values below the mass balance line (10.1% ^57^Fe) (dashed line in Figure 5). Second, the final extract in the 30 min reactors and the final three extractions in the 2 h reactors had ^57^Fe values (9.89% and 11.9% ^57^Fe, respectively) greater than those of the previously extracted aliquots (7.87% and 11.4% ^57^Fe). Here, we suspect that the exchange between transforming Fh, product phases, and solution is heterogeneous, meaning not all sites in the solid assemblage are equally accessible for exchange as the assemblage evolves. This is a feature that arises in models that allow for a separated reservoir of solids that do and do not exchange isotopes with the fluid (termed heterogeneous exchange or a “burial” model).^57,59,60^ We thus assume that as Lp and Gt form, a limited or slow back-reaction between aqueous Fe(II) and the transformed solids occurs, and a portion of ^57^Fe from solution is “buried” within the first solids that recrystallize, resulting in ^57^Fe enrichment in these solids and a depletion of the solution. As the solid assemblage continues to evolve, the zonation of ^57^Fe into the earliest dissolving particles during our 0.1 HCl extraction and subsequent “burial” of the ^57^Fe into crystalline products is consistent with the initial formation of nanostructured Lp-like particles. These particles are derived from a mix of underlying Fh and aqueous Fe(II)^30^ that subsequently grow to form larger crystallites that are not completely dissolved by our dilute HCl extraction. This interpretation is also consistent with the slightly higher *T*_B_^M^ derived from our Mössbauer spectral data for ^56^Fh reacted with ^57^Fe before and after XO extraction, where a small portion of ^57^Fe is being buried into more crystalline XO-inaccessible particles. The burial of ^57^Fe in Lp could explain the recent observation that ^57^Fe from solution preferentially forms Lp and ^57^Fe from Fh solids preferentially forms Gt over 24 h.^36^ Although we cannot unambiguously claim that the precursor Lp-like lamellae are in fact the seeds that grow into bulk Lp, the topological and chronological correspondence is quite striking. In addition, deposition of enriched ^57^Fe at the core of these particles through dissolution–reprecipitation, ultimately leading to a tracer burial mechanism as they transform into larger Lp crystallites, is consistent with the wave-like layer-by-layer growth observed by *in situ* TEM movies.^33^

## Conclusions

Our findings show that during the initial stages of Fe(II)-driven Fh transformation, before crystalline products appear, rapid isotope mixing leads to the emergence of a more magnetically ordered phase that may be linked to precursor phases documented in TEM studies.^33,34^ The Mössbauer signature of this precursor phase is removed by XO extraction, the same extraction used to isolate a labile form of Fe(III), the concentration of which appears to control XRD-detectable product mineral formation.^30,35,37^ Rapid isotope mixing between aqueous Fe(II) and Fh over this time period appears to result in the sequestration of a fraction of the ^57^Fe within the transforming solids, likely within the more stable and continually growing product phases. Our results are therefore strongly supportive of the idea that reactive intermediates form in the Fe(II)–Fh system through dissolution–reprecipitation and that dissolution–reprecipitation is an important pathway for the conversion of metastable Fh to crystalline Fe oxides.

We evaluated several possible explanations for these observations, including (i) that Fe(II)–Fh electron transfer and templated growth of Fe(III) at the surface change the surface structure and decrease the number of defects; (ii) that there is an increase in the Fh particle size during transformation; (iii) that a modified Fh structure^49,52^ emerges that is somehow stabilized by Fe(II); and/or (iv) that we have spectroscopically, and using extractions, measured properties of the nanostructured and more-ordered phases, such as lamellar Lp-like sheets that emerge early during transformation.^33,34^ Although more work is necessary, the most likely explanation appears that we have captured the properties of these early precursor products: intermediate nanostructured phases of Fe(III) oxyhydroxides, primarily Lp, formed during the lag phase of Fh transformation where no XRD-crystalline phases are observed.^34^ The rapid and extensive mixing of Fe atoms we observed here is not consistent with a surface phenomenon where Fe(II)–Fh ET results solely in the annealing of defects, akin to what we observed with Gt.^50,51^ Although we have no evidence for changes in Fh particle size and crystallinity, methods such as X-ray pair distribution function analysis may be able to provide more certainty in this regard^47,61,62^ and could resolve changes in Fh size from crystallographic changes during precipitation of the nanostructured forms of Lp. Further computational evaluation of the possibility for Fe(II)-stabilized intermediate Fh derivative structures^49,52^ could also be valuable.

A first step in Fh transformation involving nanostructured Lp-like intermediates might also provide an explanation for why slow transformation of Fh to predominantly Lp, but not Gt, occurs in systems where moderate concentrations of surface-active ligands, such as NOM^25,31,63^ and silicate,^64^ are present. We envision that as the reactive surface area decreases during Fh transformation, higher surface concentrations of ligands at the Lp surface would accumulate further, hindering Fh transformation to Gt. At higher ligand concentrations, it is also possible that nucleation of this Lp-like intermediate is entirely suppressed, resulting in the preservation of Fh even in the presence of Fe(II).^25,29,65^ In addition to surface ligands, the transformation of Fh (or its absence) strongly influences the availability of trace metals. For example, Fh transformation results in the initial release of trace metals, such as Zn and Ni, followed by later adsorption and incorporation into more stable goethite and hematite products.^66,67^ In part, the release of Ni and Zn has been attributed to competitive sorption by Fe(II);^66^ however, given the early emergence of Lp-like intermediates during this phase, differences in metal compatibility in Lp’s relative to Fh and Gt or hematite might also explain metal release behavior. Our work suggests that the mineralogical changes in the initial lag phase of Fh transformation by Fe(II) are critical to understanding Fh behavior in soils and sediments, particularly with regard to metal uptake and release.

## Supporting Information Available

A figure of the XRD results, a figure of the Mössbauer spectral data used to determine the blocking temperature, SEM images, further details on solution measurements, Mössbauer spectral fits, and solution and Fe isotope data supporting the results (PDF)
