# Supplementary material for: Tracking Initial Fe(II)-Driven Ferrihydrite Transformations: A Mössbauer Spectroscopy and Isotope Investigation
Source: ACS Earth Space Chem. 2023 Sep 28;7(10):1814–24. doi: 10.1021/acsearthspacechem.2c00291 (PMC10591510; doi:10.1021/acsearthspacechem.2c00291)
Supplement: Supplementary file 1 — sp2c00291_si_001.pdf [file sp2c00291_si_001.pdf]

SUPPORTING INFORMATION

Tracking initial Fe(II)-driven ferrihydrite transformation: A Mössbauer spectroscopy and isotope investigation

*Drew Latta,[1] Kevin Rosso,[2] Michelle Scherer[1]

[1] Department of Civil and Environmental Engineering/IIHR, The University of Iowa, Iowa City, IA 52242 USA

[2] Physical Sciences Division, Pacific Northwest National Laboratory, Richland, WA 99345 USA

Scanning Electron Microscopy Methods and Discussion

Scanning electron microscopy (SEM) images were collected on a Hitachi S-4800 after washing Fh solids exposed to buffer with and without Fe(II) three times with DI water. Solids were resuspended in methanol and dried on silicon wafers attached to aluminum SEM stubs with conductive carbon tape.

We observed the formation of platy and thin sheet-like features in SEM images after 20 minutes of Fh reaction with Fe(II) (arrows in **Figure S3**, images c and d). After 150 minutes, as expected based on XRD results, we observed both lepidocrocite (Lp) sheets and goethite nano-stars (**Figure S3**, e and f). In contrast, these materials were not observed when Fh was suspended in buffer alone (Figure S3, a and b). The Lp nanosheets after 20 minutes of reaction appear to have more defects or porosity and lack defined crystal faces when compared with those formed after 150 minutes of reaction, when the Lp sheets have clearly developed terminating faces.

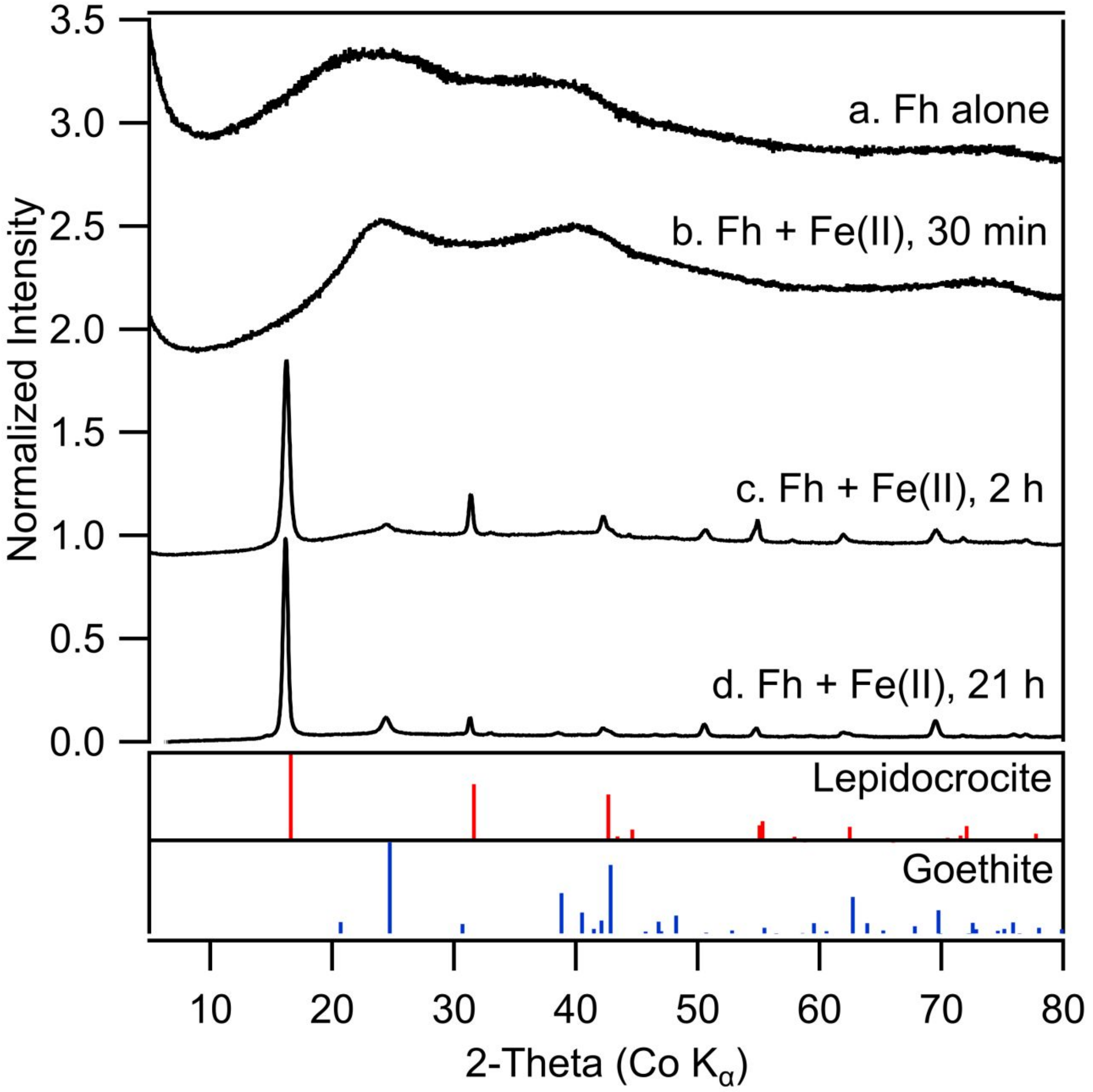

Figure S1. X-ray diffraction patterns of ferrihydrite alone in buffer (a), and ferrihydrite reacted with Fe(II) (b-d) for 30 min (b), 2 hours (c), and 21 hours (d). Conditions: 1mM ^{57}Fe(II), 10 mM ferrihydrite Fe(III), 10 mM MOPS and 10 mM NaCl at pH 7.0.

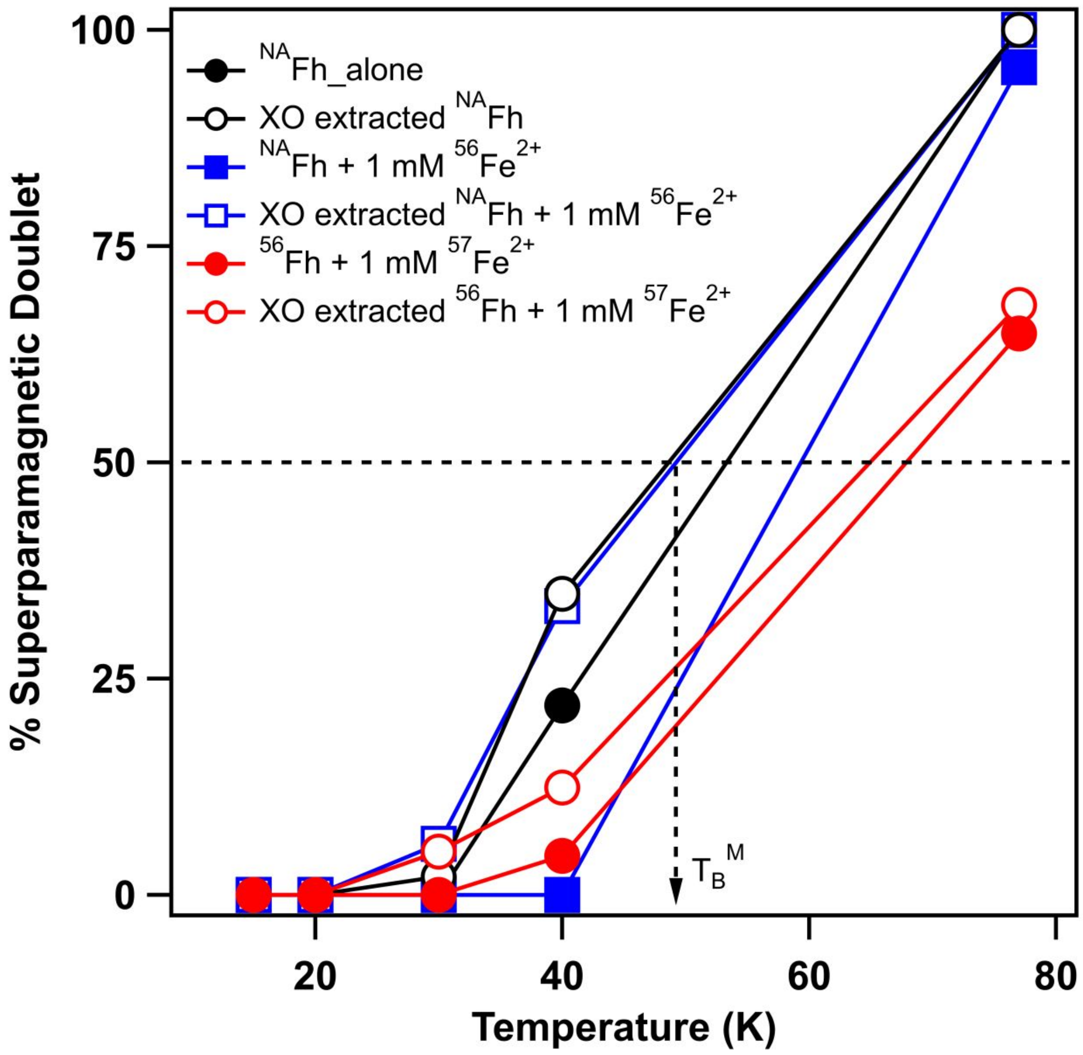

Figure S2. Spectral area (in percent) of Mössbauer spectroscopic superparamagnetic Fe(III) doublet, which is used to estimate superparamagnetic blocking temperature (T_B^M). Fitted spectral values and areas are given in **Table S2**. The dotted line shows the T_B^M estimation procedure for XO extracted ^{NA}Fh as an example.

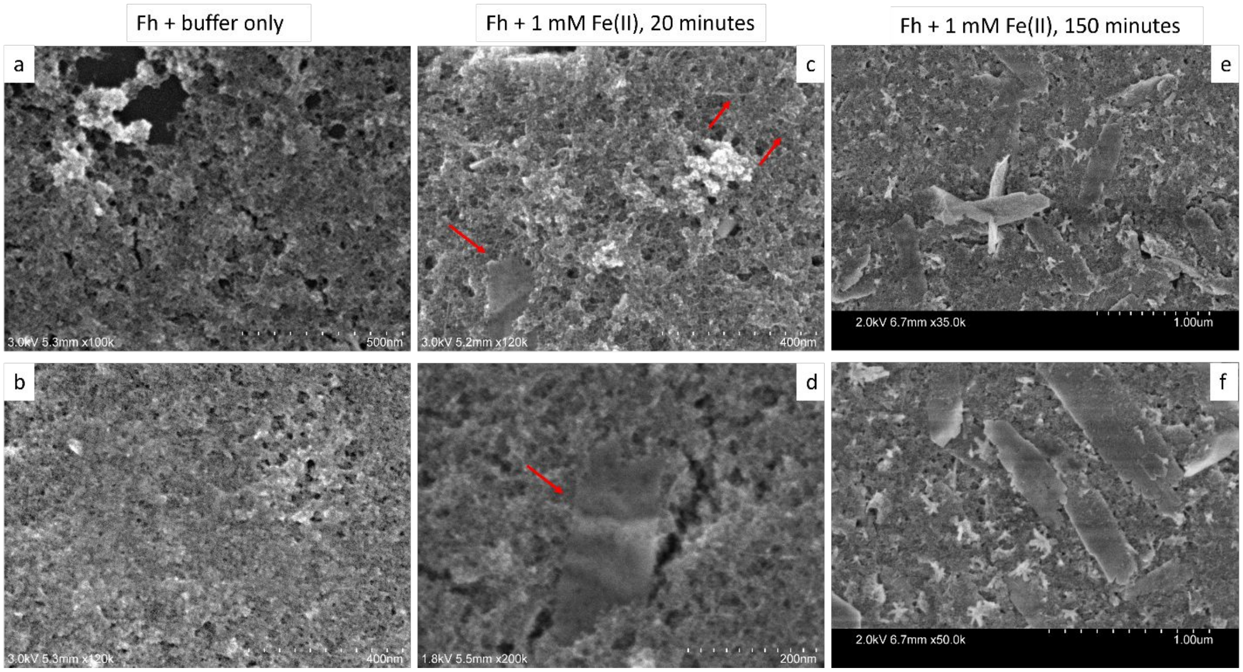

Figure S3. Scanning electron microscopy (SEM) images of Fh in buffer (a, b), Fh reacted with 1 mM Fe(II) for 20 minutes (c, d), and Fh reacted with 1 mM Fe(II) for 150 minutes. Arrows in c and d point to thin, sheet like materials formed after reaction with Fe(II). Conditions: ~10 mM Fh, 10 mM MOPS buffer, 10 mM NaCl, pH 7.0.

Table S1. Solution measurements of ferrihydrite and ferrihydrite reacted with Fe(II).

Sample	Time (min)	pH	Aqueous $Fe(II)_{initial}$ (mmole L^{-1})	Sorbed Fe(II) (mmole L^{-1})	Fh Fe(III) (mmole L^{-1})	Total Fe(II):Fh ratio	Sorbed Fe(II): Fh ratio	XO Extracted Fe(III) (mmole L^{-1})
^{NA}Fh	-	7.00	-	-	~10 mM	-	-	-
XO extracted ^{NA}Fh	-	7.00	-	-	~10 mM	-	-	0.022
^{NA}Fh + $^{56}Fe(II)$	20	7.06	1.22	0.595	8.39	0.145	0.071	-
XO Extracted ^{NA}Fh + $^{56}Fe(II)$	20	7.00	1.02	0.618	9.35	0.109	0.066	0.267
^{NA}Fh + $^{56}Fe(II)$	120	6.93	1.00	0.335	7.54	0.134	0.044	-
^{56}Fh	-	7.00	-	-	~10 mM	-	-	-
XO extracted ^{56}Fh	-	7.00	-	-	~10 mM	-	-	0.013
^{56}Fh + $^{57}Fe(II)$	20	7.01	0.744	0.273	5.90	0.126	0.046	
XO extracted ^{56}Fh + $^{57}Fe(II)$	20	6.99	1.34	0.408	6.35	0.211	0.0644	0.191
	Time (min)	pH	Aqueous $Ni_{initial}$ (mmole L^{-1})	Sorbed Ni (mmole L^{-1})	Fh Fe(III) (mmole L^{-1})	Ni:Fh ratio	Sorbed Ni Fh ratio	
^{NA}Fh + Ni	20	7.07	1.09	0.262	6.78	0.161	0.039	-

Table S2. Mössbauer fit parameters of ferrihydrite and ferrihydrite reacted with Fe(II).

			Mössbauer spectral fit parameters					
Sample	Time (min)	Mössbauer temperature (K)	Component	CS[a] (mm s^{-1})	QS[b] (mm s^{-1})	|H|[c] (Tesla)	Std(H) (Tesla) or Std(QS) (mm s^{-1})[d]	Area (%)
^{NA}Fh	0	77	Fh doublet	0.46 (0.004)[e]	0.71 (0.006)[e]	-	0.34	100
		40	Fh doublet	0.46 (0.006)	0.83 (0.03)	-	0.45	21.9 (3.1)[e]
			Fh sextet	0.44 (0.02)	-0.024 (0.02)	14.7	12.6	78.1 (3.1)
		30	Fh sextet	0.42 (0.04)	-0.029 (0.03)	28.6	13.0	100
		22	Fh sextet	0.47 (0.01)	-0.004 (0.009)	38.8	9.2	100
		15	Fh sextet	0.47 (0.002)	-0.01 (0.002)	43.1	6.78	100
^{NA}Fh + ^{56}Fe(II)	20	77	Fh doublet	0.47 (0.002)	0.82 (0.003)	-	0.61	95.7 (0.4)
			Fe^{2+} doublet	1.27 (0.03)	2.78 (0.06)	-	0.38	4.3 (0.4)
		40	Fh sextet	0.51 (0.05)	0.03 (0.05)	21.8	15.4	100
		30	Fh sextet	0.47 (0.01)	-0.02 (0.009)	34.5	12.2	100
		22	Fh sextet	0.47 (0.005)	-0.02 (0.004)	41.0	9.21	100
		15	Fh sextet	0.47 (0.005)	-0.01 (0.005)	44.4	6.77	100
^{NA}Fh + ^{56}Fe(II)	120	30	Goethite	0.54 (0.02)	-0.17 (0.02)	49.9	0.96	6.8 (1.1)
			Lepidocrocite	0.49 (0.004)	0.02 (0.004)	44.9	0.77	28.4 (0.9)
			Fh	0.47 (0.01)	-0.03 (0.02)	36.7	11.3	64.8 (1.1)
		15	Goethite	0.53 (0.01)	-0.15 (0.01)	49.9	1.1	8.2 (1.2)
			Lepidocrocite	0.48 (0.004)	0.030 (0.004)	45.0	0.78	27.7 (1.7)
			Fh	0.50 (0.004)	-0.045 (0.004)	36.5	11.5	64.0 (1.8)
^{56}Fh[f]	0		n/a[a]	n/a	n/a	n/a	n/a	n/a
^{56}Fh + ^{57}Fe(II)	20	77	Fh doublet	0.46 (0.004)	0.74 (0.004)	-	0.38	64.9 (1.1)
			Fh collapsed sextet	0.57 (0.05)	0	14.9	10.95	31.0 (0.6)
			Fe^{2+} doublet	1.16 (0.04)	3.06 (0.07)	-	0.39	4.1 (1.1)
		40	Fh doublet	0.47 (0.01)	0.88 (0.06)	-	0.51	4.5 (1.6)
			Fe^{2+} doublet	1.17 (0.07)	3.38 (0.10)	-	0.82	2.2 (0.2)
			Fh sextet	0.48 (0.006)	-0.04 (0.006)	26.3	14.5	93.3 (1.6)
		30	Fe^{2+} doublet	1.17 (0.07)	3.71 (0.13)	-	0.3	0.72 (0.17)
			Fh sextet	0.48 (0.007)	-0.03 (0.006)	34.9	12.3	99.3 (0.17)
		22	Fh sextet	0.48 (0.003)	-0.017 (0.003)	40.8	9.69	100
		15	Fh sextet	0.47 (0.003)	-0.015 (0.003)	44.4	7.63	100
XO extracted ^{NA}Fh alone	-	77	Fh doublet	0.46 (0.003)	0.72 (0.005)	-	0.38	100
		40	Fh doublet	0.47 (0)	0.73 (0)	-	0.37	34.8 (0)
			Fh collapsed sextet	0.47 (0)	0.001 (0)	12.3	11.2	65.2 (0)
		30	Fh doublet	0.44 (0.06)	0.68 (0.09)	-	0.34	2.1 (0.74)
			Fh collapsed sextet	0.46 (0.05)	0.001 (0.04)	24.6	15.0	97.9 (0.74)
		22	Fh sextet	0.47 (0.005)	-0.006 (0.005)	36.0	12.7	100
		16	Fh sextet	0.48 (0.005)	-0.01 (0.005)	42.2	7.7	100

Table S2 (continued)

Sample	Time (min)	Mössbauer temperature (K)	Component	CS[a] (mm s^{-1})	QS[b] (mm s^{-1})	|H|[c] (Tesla)	Std(H) (Tesla) or Std(QS) (mm s^{-1})[d]	Area (%)
XO extracted ^{NA}Fh + ^{56}Fe(II)	20	77	Fh doublet	0.46 (0.004)	0.73 (0.005)	-	0.43	100
		40	Fh doublet	0.47 (0.004)	0.73 (0.02)	-	0.39	33.4 (3.5)
			Fe^{2+} doublet	1.25 (0.05)	3.02 (0.12)	-	0.45	2.8 (0.8)
			Fh sextet	0.46 (0.03)	-0.024 (0.03)	16.2	14.5	63.9 (3.5)
		30	Fh doublet	0.47 (0.04)	0.74 (0.24)	-	0.47	5.9 (2.7)
			Fe^{2+} doublet	1.26 (0.06)	3.16 (0.01)	-	0.31	1.6 (0.4)
			Fh sextet	0.43 (0.03)	-0.035 (0.02)	26.6	14.8	92.5 (2.7)
		22	Fe^{2+} doublet	1.15 (0.06)	3.55 (0.12)	-	0.31	1.9 (0.5)
			Fh sextet	0.48 (0.02)	-0.019 (0.01)	36.7	11.8	98.1 (0.5)
		16	Fh Sextet	0.48 (0.003)	-0.013 (0.003)	41.3	9.3	100
XO Extract ^{56}Fh + ^{57}Fe(II)	20	77	Fh doublet	0.47 (0.03)	0.77 (0.03)	-	0.48	68.2 (0.8)
			Fe^{2+} doublet	1.25 (0.02)	3.17 (0.03)	-	0.45	6.2 (0.4)
			Fh collapsed sextet	0.52 (0.03)	0[g]	13.3	10.0	25.6 (0.8)
		40	Fh doublet	0.49 (0.002)	0.87 (0.003)	-	0.45	12.4 (0.2)
			Fe^{2+} doublet	1.30 (0.004)	3.13 (0.007)	-	0.46	5.6 (0.08)
			Fh sextet	0.48 (0.005)	-0.035 (0.006)	21.8	15.5	81.9 (0.2)
		30	Fh doublet	0.52 (0.03)	0.75 (0.08)	-	0.32	5.0 (0.2)
			Fe^{2+} doublet	1.28 (0.01)	3.17 (0.03)	-	0.44	1.5 (1.0)
			Fh sextet	0.48 (0.01)	-0.030 (0.009)	28.7	14.8	93.6 (1.0)
		22	Fh Sextet	0.48 (0)	-0.024 (0)	36.7	12.2	95.2 (0)
			Fe^{2+} doublet	1.31 (0)	3.17 (0)	-	0.52	4.8 (0)
		18	Fh Sextet	0.48 (0.003)	-0.02 (0.003)	40.8	9.94	95.1
			Fe^{2+} doublet	1.33 (0.02)	3.14 (0.04)	-	0.62	4.9
^{NA}Fh + Ni	20	15	Fh sextet	0.47 (0.003)	-0.01 (0.003)	42.4	6.86	100

[a] Center shift
[b] Quadrupole splitting for doublets and quadrupole shift for sextets
[c] Average Hyperfine field
[d] Standard deviation of the Voigt profile for the hyperfine field or quadrupole splitting parameters, respectively.
[e] Values in parethesis are the standard error (1σ) of CS, QS, and Area (%) derived from fitting.
[f] No Mössbauer effect is observed for ^{56}Fe-ferrihydrite.
[g] Paramter fixed during fitting

Table S3: Fe mass and isotope compositions during $^{57}Fe(II)$ reaction with ferrihydrite over various times. Data shown in Figure 2.

Time Reacted with Fe(II) (h)	Fraction	Fe(II) (μmol)	Fe(III) (μmol)	Total Fe (μmol)	^{57}Fe percent (%)	^{57}Fe total mass (μmol)
0	aqueous	15.95 (0.05)	-	-	94.13 (0.0018)	15.01 (0.02)
	solids	-	148.8 (3.35)	-	2.14 (0.003)	3.19 (0.08)
0.35	aqueous	10.10 (0.20)	-	-	22.64 (0.35)	2.29 (0.08)
	PIPPS extract	4.28 (0.37)	0.54 (0.01)	4.81 (0.39)	16.27 (0.50)	0.78 (0.08)
	residual	-	-	137.0 (8.27)	9.41 (0.01)	12.89 (0.79)
2.1	aqueous	10.08 (0.13)	-	-	7.95 (0.34)	0.80 (0.04)
	PIPPS extract	4.13 (0.25)	0.44 (0.03)	4.58 (0.27)	7.57 (0.06)	0.35 (0.02)
	residual	-	-	131 (1.77)	10.23 (0.04)	13.44 (0.17
21	aqueous	11.48 (0.13)	-	-	8.33 (0.10)	0.96 (0.02)
	PIPPS extract	2.05 (0.10)	0.21 (0.01)	2.26 (0.01)	8.70 (0.08)	0.20 (0.01)
	residual	-	-	145.5 (9.87)	10.39 (0.14)	15.12 (0.89)

[a] Values in parentheses represent one standard deviation of triplicate reactors.

Table S3: Fe mass and isotope compositions in each fraction during the extractions of Fh reacted with ^{57}Fe(II) for 0.5 and 2 hours. Data shown in Figure 5.

Time Reacted with Fe(II) (h)	Fraction	Fe(II) (μmol)	Fe(III) (μmol)	Total Fe (μmol)	^{57}Fe percent (%)	^{57}Fe total mass (μmol)	$\Delta^{i}f^{57}$Fe Incremental ^{57}Fe mass in extract (μmol)	$^{i}f^{57}$Fe Incremental ^{57}Fe fraction in extraction step (%)	Sum of ^{57}Fe mass (μmol)	Percent of Fe mass recovered (%)
0.7	initial aq.	14.8 (0.2)[a]	-	14.8 (0.2)	95.5 (0.43)	14.1 (0.21)	-	-	-	-
	initial solids	-	159.0 (0.7)	159.0 (0.7)	2.12 (0.40)	3.37 (0.02)	-	-	-	-
	final aqueous	8.04 (1.8)	-	8.04 (1.8)	20.3 (0.91)	1.63 (0.35)	1.63 (0.35)	20.3 (0.41)	15.2	95.9
	PIPPS extract	4.01 (0.02)	0.71 (0.02)	4.72 (0.04)	15.7 (0.07)	0.74 (0.003)	0.74 (0.003)	15.7 (0.15)		
	**0.1 M HCl** 5 min	-	-	19.5 (0.96)	12.4 (0.48)	2.41 (0.11)	0.30 (0.01)	12.4 (0.08)		
	10 min	-	-	28.6 (1.11)	11.4 (0.58)	3.25 (0.13)	0.84 (0.07)	9.30 (0.24)		
	15 min	-	-	37.6 (0.91)	10.7 (0.47)	4.03 (0.10)	0.78 (0.05)	8.63 (0.10)		
	22 min	-	-	45.7 (0.50)	10.3 (0.23)	4.71 (0.05)	0.67 (0.06)	8.30 (0.30)		
	32 min	-	-	58.6 (1.23)	9.73 (0.65)	5.70 (0.12)	1.00 (0.07)	7.73 (0.08)		
	50 min	-	-	75.9 (1.99)	9.18 (1.10)	6.96 (0.18)	1.26 (0.06)	7.28 (0.03)		
	115 min	-	-	121.8 (2.47)	8.44 (1.38)	10.3 (0.21)	3.32 (0.07)	7.22 (0.06)		
	Residual	-	-	147.6 (3.33)	8.69 (1.87)	12.8 (0.26)	2.55 (0.25)	9.89 (0.23)		
2	initial aq	15.8 (0.1)	-	15.8 (0.1	94.4 (0.06)	14.9 (0.06)	-	-	-	-
	initial solids	-	162.4 (2.1)	162.4 (2.1)	2.08 (0.00)	3.38 (0.04)	-	-	-	-
	final aqueous	7.64 (0.38)	-	7.64 (0.38)	9.04 (0.15)	0.691 (0.04)	0.691 (0.04)	9.04 (0.15)	16.7	91.5
	PIPPS extract	5.90 (0.32)	0.13 (0.08)	6.03 (0.40)	8.71 (0.04)	0.525 (0.03)	0.525 (0.03)	8.71 (0.04)		
	**0.1 M HCl** 12 min	-	-	76.1 (4.70)	8.24 (0.08)	6.26 (0.34)	0.516 (0.02)	8.24 (0.08)		
	30 min	-	-	81.9 (3.94)	8.45 (0.11)	6.96 (0.27)	0.584 (0.02)	8.45 (0.11)		
	60 min	-	-	81.8 (3.22)	8.63 (0.11)	7.06 (0.21)	0.793 (0.14)	14.1 (1.2)		
	1 M HCl extract	-	-	3.13 (0.13)	11.4 (0.10)	0.36 (0.01)	0.041 (0.001)	11.4 (0.10)		
	Residual	-	-	68.5 (2.67)	11.9 (0.23)	8.13 (0.16)	0.964 (0.009)	11.9 (0.23)		

[a] Values in parentheses represent one standard deviation of triplicate reactors.
